# If there is LV myocardial fibrosis should we expect to find RV myocardial fibrosis? A cardiovascular MRI study

**DOI:** 10.1186/1532-429X-14-S1-P151

**Published:** 2012-02-01

**Authors:** Robert W Biederman, Saundra Grant, Ronald B Williams, Vikas K Rathi, Mark Doyle, June Yamrozik

**Affiliations:** 1 Allegheny General Hospital, Pittsburgh, USA

## Summary

In HCM, LGE defined LV fibrosis is commonplace and generally not considered in the RV. However, by CMR-LGE it may be more commonplace than expected.

## Background

CMR has become the leading technique to detect hypertrophic cardiomyopathy (HCM) providing complete coverage of both ventricles with high spatial resolution. Late gadolinium enhancement (LGE) accurately identifies regions of myocardial fibrosis. Via CMR, innumerable studies have established LVH as the predominant phenotypic expression. It is well known that myocardial fibrosis can occur in patients with HCM and is independently linked to worse prognosis than those without fibrosis by CMR. The genotypic expressions would appear to affect the entire heart yet, conventionally; descriptions have been limited to the LV, likely due to the inability to image the smaller, thinner RV. Thus, little information is available about the RV in HCM. We hypothesize there may be RV involvement in HCM when incorporating CMR analysis for RV hypertrophy and fibrosis.

## Methods

Review of all patients referred for HCM was performed. SSFP/LGE was used to diagnose patients with HCM, using gadolinium administration (MultiHance, 0.15mmol/kg, Bracco Diagnostics, Princeton, NJ). Post-injection LGE images were obtained via T1-weighted, IR preps. Regions of myocardium with abnormally high signals (>2SD) were designated as fibrotic. LV/RVMass Index was calculated.

## Results

Via 99 patients referred for HCM from 2006-2011, 28 (28%) were confirmed to be HCM via CMR. Image quality was judged excellent in 27/28 (97%). The mean LVMI was 97±40gm2 (> 2SDabove normal) while the mean RVMI was 22±5 gm2 (>1SD > normal). All patients met formal LVH criteria while 17/28 (61%) met RVH criteria. LV fibrosis was evident in 24/28 (86%) while most, 19/28 (67%), also had evidence for RV fibrosis. No patients with RV fibrosis had absent LV fibrosis. In neither the LV nor RV did the degree of hypertrophy predict the likelihood for fibrosis (98±38 vs.84±26g/m2 and 22±24 vs.21±25g/m2, respectively).

## Conclusions

The high frequency of RV fibrosis in the setting of HCM is unforeseen presumably as this phenomenon is not anticipated. However, given the genetic abnormalities, there is no reason to expect the phenotypic expression should be limited to just the LV. Interestingly, similar for LVH, the degree of RVH had little predictive power to define fibrosis.

## Funding

Internal.

